# The Spritztube: A New Device for the Extraglottic Intubation of Rabbits

**DOI:** 10.3390/ani13010156

**Published:** 2022-12-31

**Authors:** Carlotta Lambertini, Annamaria Grandis, Margherita De Silva, Ilaria Anna Cassano, Stefano Checcacci Carboni, Noemi Romagnoli

**Affiliations:** 1 Department of Veterinary Medical Sciences, Alma Mater Studiorum-University of Bologna, Via Tolara di Sopra 50, Ozzano Emilia, 40064 Bologna, Italy; 2 Veterinarian Private Practitioner, 20067 Milan, Italy; 3 Anaesthesia and Reanimation, Hospital of Vicenza, Az ULSS 8 Berica, 36100 Vicenza, Italy

**Keywords:** anaesthesia, extraglottic intubation, rabbits, spritztube

## Abstract

**Simple Summary:**

Several factors contribute to increasing the peri-anaesthetic risk in rabbits; above all, the airway management is challenging due to their peculiar anatomic characteristics. In human anaesthesia, the Spritztube (ST), an extraglottic airway device, has recently been developed for airway management. In the present study, an anatomical evaluation of rabbit cadavers was first carried out with the aim of designing a specific ST for rabbits. In the second phase of the study, the STs designed were evaluated on privately owned rabbits undergoing general anaesthesia to test their feasibility and safety regarding airway management in this species. The results demonstrated that the correct positioning of the ST can be completed in 43 ± 21.4 s with an average of 2.1 ± 1 attempts. Respiratory distress during ST positioning was the only complication observed in one rabbit. In conclusion, STs specifically designed for rabbits can be used as a supraglottic airway device for the maintenance of isoflurane anaesthesia in spontaneously breathing animals.

**Abstract:**

The Spritztube (ST) is an extraglottic airway device developed for humans. The aim of the study was to design an ST for rabbits and to evaluate its feasibility. The study was divided into two phases. Phase I: anatomical study on 12 rabbit cadavers to design 2 STs (8 and 10 Ch, external diameter) for rabbits. Phase II: fourteen privately owned rabbits were anaesthetised, and intubation was attempted using a ST. Tube size, the method for confirming the correct positioning, the number of attempts, the time needed for the correct positioning of the ST and complications were recorded. The ST placement was feasible in all rabbits. The positioning of the ST was completed in 2.1 ± 1 attempts in 43 ± 21.4 s. A correct placement was confirmed by the visualisation of the proximal cuff at visual inspection of the oral cavity (14/14), by the detection of the airflow (9/14 rabbits) and by the visualisation of a capnographic wave (14/14 rabbits). Only one rabbit developed respiratory distress after the ST placement. The results of the present study allowed designing a ST specific for rabbits which was used a supraglottic airway device for the maintenance of isoflurane anaesthesia in spontaneously breathing rabbits.

## 1. Introduction

Rabbits are at high risk of developing peri-anaesthetic complications, one of which is the overall incidence of anaesthetic-related death in this species of up to 1.38%, with this being higher than that reported for other small companion animals [1].

Several factors contribute to increasing the peri-anaesthetic risk in rabbits; above all, the airway management is challenging due to their peculiar anatomic characteristics. In fact, rabbits are characterised by a temporomandibular joint with a limited range of motion, a long and narrow pharynx with a large tongue and big incisors which hinder the visualisation of the larynx [2]. In addition, intubation attempts might induce laryngospasms or tracheal injury [3]. In the case of severe damage, airway obstruction with subsequent respiratory complications up to sudden death have been reported [3].

With the aim of overcoming these difficulties, extraglottic airway devices have been investigated for rabbit airway management as an alternative to orotracheal tubes [4,5,6]. Several authors have evaluated the feasibility of insertion and the safety of laryngeal mask airway devices in rabbits [5,6,7]. The laryngeal mask is an airway device originally designed to be used for humans, consisting of a mask attached to a tube which is blindly inserted into the hypopharynx. In rabbits, this device has some advantages over orotracheal intubation, namely, easier and faster insertion with less incidence of laryngeal and tracheal tissue injuries [8,9]. In addition, a supraglottic airway device has been designed specifically for rabbits (V-Gel^®^) by adapting the human supraglottic airway device i-gel to obtain easy self-positioning over the laryngeal inlet of rabbits [10,11].

The Spritztube (ST) is an extraglottic airway device, which has recently been developed for airway management in human patients and was first described by De Rosa and colleagues [12]. The ST is characterised by the presence of a distal cuff which is blindly placed in the oesophagus to seal it and a proximal cuff which seals the epiglottis, allowing patient ventilation. The two cuffs are maintained aligned with the aid of a central rigid guide. After its introduction and after patient stabilisation, the same device can be turned into a regular endotracheal tube by advancing it into the trachea under laryngoscopic guidance [12,13,14].

The aim of the study was to design an ST suitable for rabbits. The study was therefore divided into two phases. Phase I was conceived as an anatomical study on rabbit cadavers in order to obtain measurements of the upper airway for developing a prototype of the ST for this species. In phase II, an in vivo study was carried out to evaluate the ease of insertion and the safety of the ST in rabbits undergoing general anaesthesia.

## 2. Materials and Methods

### 2.1. Phase I

Twelve fresh privately owned rabbit cadavers (6 females and 6 males), with a mean body weight of 3.1 ± 2.1 kg, were included in the study. All the animals died from reasons unrelated to the upper respiratory and digestive tracts and were intended for cremation. Each rabbit was placed in the left lateral recumbency with the head and the neck at an angle as close as possible to the physiological position. The nostrils were blocked with gauze, while the lips were almost completely clamped with surgical forceps after inserting a plastic cannula in the mouth. According to the procedure described elsewhere [15], the cast of the upper respiratory and digestive tracts was then obtained by means of the injection of 20 gr of polyurethane foam (diphenylmethane-4,4-diisocyanate; Soudafoam^®^ - Soudal N.V., Turnhout, Belgium), diluted with 5 mL of acetone to prevent its over-expansion and with the addition of a few drops of yellow nitro dye (PebeoCeramic^®^; Gemenos, France). When the foam began to come out of the mouth, the injection was interrupted, the cannula was removed, and the lips were completely tightened. After finishing the casting procedure, the specimens were stored at 4 °C for 3 h to allow the foam to harden. A stepwise dissection of the lateral regions of the head and the neck was carried out starting from the skin. This allowed exposure of the pharynx, larynx, trachea, oesophagus and the mouth cavity, after the removal of the ramus and the caudal part of the body of the left mandible.

**Figure 1:**
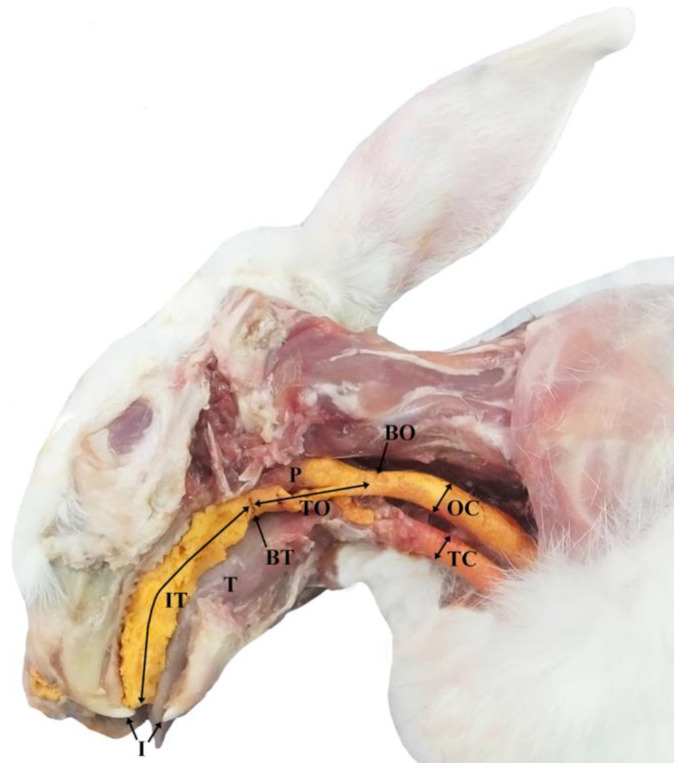
Anatomical preparation of a rabbit head. The anatomical preparation was obtained after the removal of the ramus and the caudal part of the body of the left mandible. The yellow colour indicates the cast of the upper respiratory and digestive tracts obtained by the injection of polyurethane foam. I—incisors; T—tongue; P—pharynx; BO—beginning of the oesophagus; IT—distance between the incisors and the base of the tongue; TO—distance between the base of the tongue and the beginning of the oesophagus; OC—oesophageal calibres; TC—tracheal calibres.

Once the cast was exposed, the following measurements were taken and recorded: the distance between the incisors and the base of the tongue (IT) and the distance between the base of the tongue and the beginning of the oesophagus (TO) using a measuring tape. In addition, the oesophageal (OC) and tracheal (TC) calibres were evaluated (Figure 1).

### 2.2. Phase II

Fourteen privately owned rabbits (7 males and 7 females) were included in the study. Only rabbits undergoing scheduled surgical procedures with general anaesthesia were included in the study. The rabbits were considered clinically healthy based on physical examination. The owner consent was obtained before the anaesthesia.

#### Anaesthetic Protocol

The rabbits were premedicated intramuscularly with alfaxalone (3–5 mg/kg; Alfaxan; Jurox Pty Ltd, Dublin, Ireland), xylazine (2–4 mg/kg; Rompun, Bayer, Milan, Italy) and ketamine (3 or 5 mg/kg; Nimatek, Dechra, Torino, Italy) mixed in the same syringe for injection and administered between the semimembranosus and semitendinosus muscles. Concurrently, the rabbits were preoxygenated with 100% oxygen administered using a face mask at 3 L/min, until intubation was attempted. After the onset of sedation, an intravenous catheter was placed in the marginal auricular vein after the trichotomy and aseptic preparation of the area. In addition, before induction, the following measurements were taken using a measuring tape, maintaining the rabbits in the lateral recumbency: the distance between the naris and the external projection of the thoracic inlet (NT) in cm and the distance between the naris and the external projection of the cranial border of the larynx (NL). 

General anaesthesia was induced with 5% isoflurane in 100% oxygen delivered using a facemask 10 to 15 minutes after premedication. The placement of the ST was attempted, when the palpebral reflex, the ear-pinch reflexes and the mandibular jaw tone were lost. The two cuffs of the ST were checked for leaks before insertion.

The ST was introduced blindly with the rabbit placed in the lateral recumbency and the head hyperextended. The operator positioned his/her non-dominant hand behind the mandibular angle. The tube was then advanced through the incisors towards the lateral wall of the pharynx with the dominant hand, until the operator felt the tip of the device reaching the oesophageal lumen below the index finger of the non-dominant hand. At that point, the distal oesophageal cuff was inflated, and the operator verified that the inflated oesophageal cuff was positioned caudally beyond the mandibular angle using percutaneous palpation. The central guide was then removed. The proximal cuff was inflated, and the ST was gently drawn back until the cuff stopped at the base of the tongue. Both the distal and the proximal cuffs were inflated using a syringe until resistance was felt upon insufflation, and the total amount of mL inflated in each cuff were recorded. The end of the ST was attached to a side-stream capnogram. A correct ST placement was confirmed by applying one of the following criteria: direct visualisation of the proximal cuff by opening the oral cavity, evaluation of the airflow movement or a normal capnographic wave. The tube size, the number of attempts necessary for the correct ST placement and the overall time needed for achieving the correct positioning were recorded. In addition, the death space of the ST, defined as the distance between the incisors and the machine end of the ST, was measured using a measuring tape. As soon as the correct placement of the ST was confirmed, the animals were attached to a Jackson-Rees modified Ayre's T-piece breathing system, and isoflurane was delivered in 100% oxygen. The isoflurane was initially delivered at 1.5%, and then during the procedure, the vaporiser was adjusted according with the anaesthetic depth.

The safety of the technique was evaluated based on the following criteria: the presence or absence of gross lesions or bleeding from the oral cavity, the presence or absence of cyanosis of the tongue and/or the presence or absence of respiratory distress. In the case of complications during the ST placement, any attempt at inserting the ST ceased, and a face mask was used for maintenance of general anaesthesia. 

For additional confirmation of the correct placement of the ST, X-rays scans of the head and the cervical tract and computed tomography (CT) (Philips Brilliance CT, Philips Medical Systems Technologies Ltd., Haifa, Israel) were performed in one animal each. 

Soon after intubation and every five minutes up to the end of the procedure, the following variables were evaluated: heart rate (HR) from an electrocardiogram; peripheral haemoglobin oxygen saturation (SpO2) using a pulse oximeter applied on the tongue; body temperature (T°) using a rectal probe; respiratory rate (RR), fraction of expired CO_2_ (FE’CO_2_) using a calibrated side stream gas analyser; and isoflurane %. In addition, non-invasive blood pressure (NIBP) was evaluated using a Doppler device (MiniDop) by placing the probe on the metacarpal artery. At the completion of the first evaluation, the rabbits were positioned in the recumbency required for the surgery and the surgical procedure commenced.

At the end of the surgical procedure, isoflurane was discontinued, and atipamezole was administered IM at 0.1 mg/kg. When the rabbits regained the palpebral reflex, the ST was removed and checked for blood or damage. The rabbits were closely monitored until fully awake. 

### 2.3. Statistical Analysis 

Data distribution was tested using the Shapiro−Wilk test. Normally distributed data were reported as mean and standard deviation (SD); non-normally distributed data were reported as median and range. The categorical data were reported as proportions. Analyses were carried out using MedCalc 6.3 (MedCalc computer software).

## 3. Results

### 3.1. Phase I

The mean IT and TO lengths were 5.3 ± 0.8 cm and 2.2 ± 0.8 cm, respectively. The mean EC length was 0.7 ± 0.1 cm, and the mean CT length was 0.9 ± 0.3 cm.

Based on these results, two ST were chosen to be used in the clinical part of the study: an 8 Ch and a 10 Ch device (Figure S1). 

### 3.2. Phase II

Thirteen rabbits were involved in the entire period of the study. One rabbit was removed from the study, because it developed respiratory distress after the first attempt at the ST placement. The ST was therefore removed, and the respiratory distress disappeared. The repositioning of the device was not feasible due to an insufficient anaesthetic depth, and therefore, general anaesthesia was maintained using a face mask. Of the remaining animals, the mean age was 9.4 ± 4.7 months, and their mean body weight was 1.6 ± 0.5 kg. The rabbits included in the study belonged to the following breeds: Dwarf rabbits (*n* = 7), Mini lop (*n* = 5) and Champagne Argent (*n* = 1). All the rabbits underwent ovariectomy or orchiectomy except for one, which underwent tooth trimming. The mean NT length was 9.7 ± 0.9 cm, and the mean NL length was 6.1 ± 1.4 cm.

The ST placement was feasible in all the remaining rabbits. Eight rabbits were intubated in the right lateral recumbency, and six were in left lateral recumbency. In 11 rabbits, a 10 Ch ST was used, while in 3 rabbits an 8 Ch ST was used. An average of 2.1 ± 1 attempts were necessary for completing the ST placement. Overall, a mean of 43 ± 21.4 s was necessary to complete the correct positioning of the ST. The correct positioning of the ST was confirmed by the direct visualisation of the proximal cuff at a visual inspection of the oral cavity (14/14), by the detection of the airflow in 9/14 rabbits and by the visualisation of a correct capnographic wave (14/14 rabbits). The oesophageal cuff was inflated with a median of 5 mL (range: 2–6 mL), and the proximal cuff was inflated with a median of 5 mL (range: 2–6 mL).

**Figure 2:**
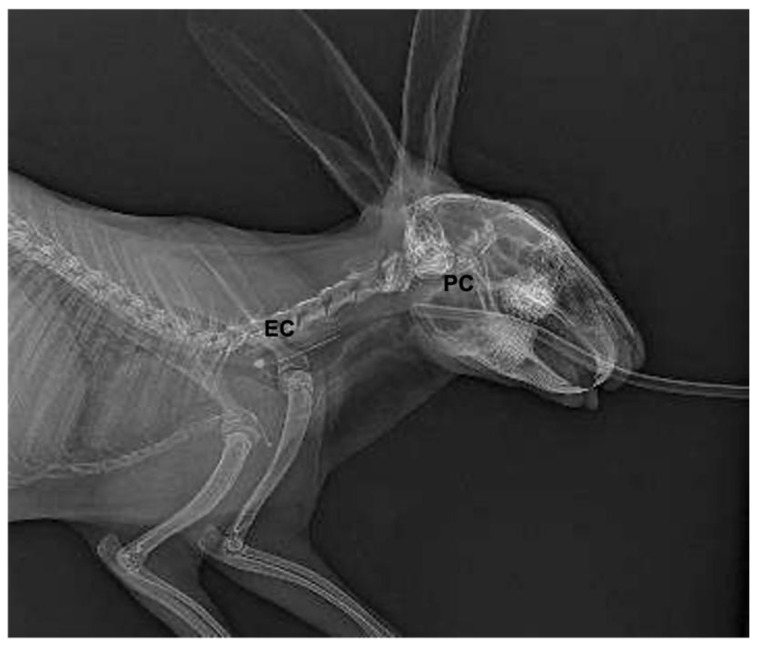
Radiographic image of a rabbit with the ST inserted. EC—oesophageal cuff within the oesophageal lumen; PC—proximal cuff in the oral cavity.

**Figure 3:**
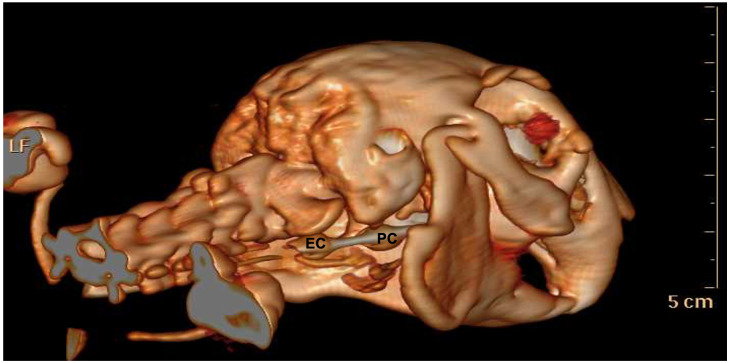
Tomographic image of a rabbit with the ST inserted. EC—esophageal cuff within the esophagus; PC—proximal cuff in the laryngeal vestibule (figure obtained from the archive of the Diagnostic image service of the Department of Veterinary Medical Sciences, University of Bologna).

With the aim of confirming the correct positioning, an X-ray scan was taken in one rabbit, and a CT scan was performed in another rabbit. In detail, at the X-ray evaluation, the oesophageal cuff was detectable in the oesophagus as a circular radiolucent structure close to the thoracic inlet (Figure 2). The proximal cuff appeared as a radiolucent structure cranial to the ramus of the mandible. The computed tomography scans confirmed the oesophageal positioning of the oesophageal cuff and the proximal cuff at the level of the laryngeal vestibule (Figure 3). 

All the cardiorespiratory variables monitored remained within normal limits. During anaesthesia or at recovery, bleeding or gross lesions were not observed. 

In all the rabbits, recovery was uneventful, and they were discharged on the same day as soon as they were fully awake.

## 4. Discussion

In the present study, the technique for the ST placement was described for the first time. The results indicated that the ST is easy to place with minimal risk of trauma. 

Respiratory complications are common in anaesthetised rabbits [9], and the rapid control of the airways is advisable to prevent hypoventilation and hypoxemia. Endotracheal tube placement is challenging in this species. A face mask can be effective for quickly providing oxygen to the patient; however, it does not prevent airway obstruction and does not allow patient-assisted ventilation [9]. Studies regarding rabbits have highlighted the superiority of a laryngeal mask over a face mask and over an endotracheal tube [8,9]. In fact, a laryngeal mask airway device is more easily and readily placed as compared with an endotracheal tube; it also provides qualitatively equivalent surgical anaesthesia [8]. In addition, it is effective for the management of airway obstruction and for providing mechanical ventilation, thereby preventing hypercapnia and hypoxemia [9]. Finally, the supraglottic airway device minimises the risks of creating laryngotracheal trauma associated with endotracheal intubation [16]. Some authors have reported that a laryngeal mask is rapid to place and requires an average of 33 s (range: 14–38 s) for correct placement [6]. In humans, the ST has been introduced as an alternative to the laryngeal mask airway device for managing airways [13]. That study demonstrated that the insertion time and the success rate for the placement of the two devices was comparable [13]. Similarly, in the present study, the ST placement required 43 ± 21.4 s. Therefore, the ST allowed achieving airway control in a shorter period of time as compared with an endotracheal tube for which the average insertion time was reported to be 59 s (range: 29–171 s) in a previous study [6]. 

The technique for ST insertion described in the present study allowed correct positioning as confirmed by the diagnostic imaging. The positioning, however, required multiple attempts (up to 4), which is a higher rate than that reported for the laryngeal mask and endotracheal tube placement [6]. However, the ST is a new device with a particular conformation, and experienced anaesthetists may therefore also require a learning period before developing a technique for easy insertion at the first attempt. In the present study, a single expert anaesthetist attempted the ST placement. In the future, the ease of the ST placement should also be evaluated for unexperienced operators. In addition, the positioning of the ST was attempted only in the lateral recumbency. In fact, this was judged to be the more suitable positioning for advancing the device and for the confirmation of the correct placement of the cuffs through external palpation. We cannot exclude that the placement of the ST with the rabbit in the sternal recumbency would provide a more rapid placement.

Compared with other devices, one of the advantages of the ST is that it is equipped with an oesophageal cuff which seals the oesophagus, which would help in correct positioning. Previously, other authors have described that oesophageal cannulisation could be used in rabbits as guidance for preventing endotracheal tube dislodgement in the oesophagus [17]. 

The following complications were recorded in humans during the insertion of the ST: air leak, laryngospasm, obstruction after insertion, the presence of gastric insufflation and sore throat upon removal [13]. In the present study, although multiple attempts were necessary for the ST placement, gross lesions or bleeding were not observed. Respiratory distress was observed in only one rabbit. On the contrary, an elevated incidence of respiratory complications and sub laryngeal lesions have been reported in rabbits, when an endotracheal tube was used [3]. In fact, rabbits might develop respiratory rales or respiratory distress up to sudden death after endotracheal intubation [3]. In the same study, the authors reported that the necroscopic and the histologic evaluations of the trachea disclose the presence of severe mucosal congestion, haemorrhage, oedema, mild bruising, stenosis and ulceration [3,6]. The use of the laryngeal mask airway device or of other supraglottic airway devices is also not without complications [5]. In fact, when is the laryngeal mask is used in rabbits, they could develop cyanosis, haemorrhage, laryngeal oedema [5,7] or also gastric tympanism during the application of controlled ventilation [9]. Another important limitation of the use of these devices is that they can be relatively easily displaced/dislodged during surgical procedures requiring changes in the recumbency of the animal or movements of the head. When this happens, a block of the airways may develop [16]. In the present study, the ST was not secured to the head of the rabbit; however, we did not observe episodes of dislodgment. This advantage can be related not only to the lightweight of the ST, but also to the inflated oesophageal cuff, which prevent the dislodgment.

Another important advantage is associated with the use of the ST. In fact, in humans, the ST is designed to be used not only as an extraglottic airway device. In fact, as soon as patient stabilisation is achieved, the same device can be advanced into the trachea under laryngoscopic or fibreoptic guidance and can be used as a regular endotracheal tube. In addition, in rabbits, the fiberoptic or rigid (1.9 to 2.7 mm) endoscopes can be used for enabling ET insertion into the trachea by direct visualisation on the endoscopic screen [18,19]. However, the length and the conformation of the ST designed for rabbits did not allow applying this technique. In addition, these techniques involve the use of expensive materials not always available in clinical settings. However, the size of the rabbits included in the present study represented an important limitation. In fact, the insertion of the fibreoptic endoscope into the oral cavity, parallel to the ST or the inspection with a laryngoscope, would place the animal at high risk of developing lesions of the oral cavity and those of the larynx [16]. For the same reason, visualisation of the device positioning or inspection of the laryngeal structure under fibroscopic guidance was not performed. It cannot be excluded that, in larger rabbits, in which the size of the oral cavity allows the introduction of instruments, the ST tube can be additionally advanced into the trachea, thereby turning into an endotracheal tube. 

Overall, in humans, the application of a laryngeal mask is associated with a greater incidence of complications (19.1%) when compared with the insertion of an ST (6.0%) [13]. Further studies are needed to evaluate the advantages of the ST over the use of a laryngeal mask airway device or that of other supraglottic airway devices (such as the V-Gel^®^) in rabbits. There are several limitations regarding the present study. First of all, the airway seal was not evaluated. Therefore, additional studies are needed to evaluate whether the ST provides an adequate airway seal for allowing controlled ventilation. In addition, in rabbits, the laryngeal mask emits 33% more waste isoflurane emission as compared with the cuffed endotracheal tube when sampled at the oral commissure [8]. In the present study, however, the extent of anaesthetic gas pollution was not evaluated. Therefore, this aspect is still to be evaluated, since in veterinary anaesthesia, exposure to inhalant anaesthetic pollution represents an important concern.

## 5. Conclusions

In conclusion, the results of the present study allowed designing an ST-specific for rabbits which was quickly positioned and was successfully used as a supraglottic airway device for maintenance of isoflurane anesthesia in spontaneously breathing rabbits.

## 6. Patents

Euorpean patent application for the Spritz tube: EP3880282A1.

## Data Availability

Data will be available upon request.

## Supplementary Materials

The following are available online at https://www.mdpi.com/article/10.3390/ani13010156/s1, Figure S1: 3D schematic representation of the Spritztube realised for rabbits.
